# Validity of pathological diagnosis for early colorectal cancer in genetic background

**DOI:** 10.1002/cam4.5596

**Published:** 2023-02-03

**Authors:** Kenichiro Okimoto, Yosuke Hirotsu, Makoto Arai, Kenji Amemiya, Naoki Akizue, Yuki Ohta, Takashi Taida, Keiko Saito, Hiroshi Ohyama, Tomoaki Matsumura, Motoi Nishimura, Kazuyuki Matsushita, Keisuke Matsusaka, Toshio Oyama, Hitoshi Mochizuki, Tetsuhiro Chiba, Jun Kato, Jun‐ichiro Ikeda, Osamu Yokosuka, Naoya Kato, Masao Omata

**Affiliations:** ^1^ Department of Gastroenterology, Graduate School of Medicine Chiba University Chiba Japan; ^2^ Genome Analysis Center Yamanashi Prefectural Central Hospital Kofu Japan; ^3^ Department of Gastroenterology Tokyo Women's Medical University Yachiyo Medical Center Yachiyo Japan; ^4^ Division of Clinical Genetics and Proteomics, Department of Laboratory Medicine Chiba University Hospital Chiba Japan; ^5^ Department of Molecular Pathology, Graduate School of Medicine Chiba University Chiba Japan; ^6^ Department of Pathology Yamanashi Central Hospital Kofu Japan; ^7^ Chiba University Chiba Japan; ^8^ Tokyo University Tokyo Japan

**Keywords:** colorectal adenoma, colorectal cancer, next‐generation sequence, pathological diagnosis, TCGA

## Abstract

**Background:**

This study aimed to investigate the validity of pathological diagnosis of early CRC (E‐CRC) from the genetic background by comparing data of E‐CRC to colorectal adenoma (CRA) and The Cancer Genome Atlas (TCGA) on advanced CRC (AD‐CRC).

**Methods:**

TCGA data on AD‐CRC were studied in silico, whereas by next‐generation sequencer, DNA target sequences were performed for endoscopically obtained CRA and E‐CRC samples. Immunohistochemical staining of mismatch repair genes and methylation of *MLH1* was also performed. The presence of oncogenic mutation according to OncoKB for the genes of the Wnt, MAPK, and cell‐cycle–signaling pathways was compared among CRA, E‐CRC, and AD‐CRC.

**Results:**

The study included 22 CRA and 30 E‐CRC lesions from the Chiba University Hospital and 212 AD‐CRC lesions from TCGA data. Regarding the number of lesions with driver mutations in the Wnt and cell‐cycle–signaling pathways, E‐CRC was comparable to AD‐CRC, but was significantly greater than CRA. CRA had significantly more lesions with a driver mutation for the Wnt signaling pathway only, versus E‐CRC.

**Conclusions:**

In conclusion, the definition of E‐CRC according to the Japanese criteria had a different genetic profile from CRA and was more similar to AD‐CRC. Based on the main pathway, it seemed reasonable to classify E‐CRC as adenocarcinoma. The pathological diagnosis of E‐CRC according to Japanese definition seemed to be valid from a genetic point of view.

## Graphical abstract

Regarding the number of lesions with driver mutations in the Wnt and cells‐cycle–signaling pathways, Early CRC was comparable to Advanced CRC. It seemed reasonable to classify Early CRC as adenocarcinoma.

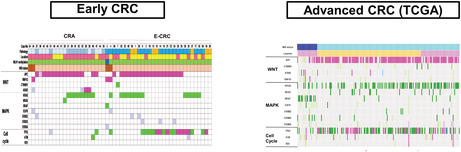

## 1 INTRODUCTION

Worldwide, colorectal cancer (CRC) is estimated to be the third and second most common cancer among men and women, respectively, with an increasing prevalence.^1^, ^2^ Although the 5‐year survival rates was reported to be 90% and 71% for localized and regionalized CRC, respectively, the 5‐year survival rate for metastatic CRC remains low at 14%.^3^ CRC is associated with several risk factors, such as a diet high in red and processed meat,^4^, ^5^ heavy smoking and alcohol intake,^6^ obesity,^7^ and a family history of CRC.^8^

Genes responsible for the carcinogenesis of CRC are already known partially, including the following: microsatellite instability (MSI) as a manifestation of mismatch repair (MMR) gene (*MLH1*, *MSH2*, *MSH6*, *PMS1*, and *PMS2*) germline deficiency,^9^, ^10^ biallelic *MUTYH* mutations leading younger‐age onset of CRC,^11^ and germline mutation of genes coding for deoxyribonucleic acid (DNA) polymerase (*POLE* and *POLD*).^12^ Somatic mutations of CRC like *KRAS*, *NRAS*, *BRAF PIK3CA*, and *ARID1A* have also been identified.^13^, ^14^, ^15^, ^16^ In recent years, The Cancer Genome Atlas (TCGA) projects analyzed big data and identified 24 somatic significantly mutated genes (SMGs) such as *SOX9* and *FAM123B*.^17^ However, these findings mainly concentrate on advanced CRC (AD‐CRC); the genetic profile of early CRC (E‐CRC) still remains to be fully clarified.

The pathological diagnosis of CRC differs between Japan and Western countries. Based on the WHO classification, CRC is defined as invading the submucosa (SM).^18^ However, in Japan, CRC is defined by a combination of nuclear and structural atypia, with or without invading the SM layer.^19^ In addition, the conception of E‐CRC in Japan including not only SM invasive cancer but also intramucosal carcinoma (IMC) does not exist in the WHO classification. And it remains unclear whether the pathological definition of E‐CRC is genetically correct or not.

As for the prognosis of E‐CRC, the detection and endoscopic removal of colonic lesions were reported to reduce the incidence and mortality of CRC by 53%.^20^ Therefore, endoscopic treatment for E‐CRC is now considered an important method to prevent CRC‐related death. Receiving these results, the prognosis between E‐CRC and localized, regionalized, or metastatic AD‐CRC seems to differ greatly, so it is important to understand the genetic characteristics of E‐CRC compared to AD‐CRC.

Thus, the aim of this study was to verify the validity of the pathological diagnosis of E‐CRC from the genetic background by comparing data on colorectal adenoma (CRA), and TCGA data on AD‐CRC.

## 2 METHODS

### 2.1 Patients and sample preparation

In this study, 15 patients with 22 lesions of CRA and 28 patients with 30 lesions of E‐CRC, according to the Japanese criteria,^19^ in Chiba University Hospital were included. We obtained neoplastic tissue samples via endoscopic mucosal resection (EMR) or fresh specimen from endoscopic submucosal dissection (ESD). DNA of neoplastic lesion was extracted with the QIAamp DNA Mini Kit (Qiagen, Hilden, Germany). DNA of buffy coats for all 43 patients of CRA and E‐CRC were also extracted according to previous reports.^21^ For AD‐CRC, 212 lesions of TCGA data were included.

### 2.2 Ethical statement

This study was approved by the bioethics committee of the Chiba University Hospital (No 805) and was implemented according to the Declaration of Helsinki. Written informed consent was obtained from all patients in this study.

### 2.3 Pathological diagnosis of CRA, E‐CRC, and AD‐CRC

Pathological diagnosis was followed by Japanese Classification of Colorectal, Appendiceal, and Anal Carcinoma ver 9.0.^19^ In short, low/high‐grade adenomas were included in CRA. IMC and SM invasive cancer were included in the E‐CRC. On the other hand, in the TCGA data, UICC T3/T4a/T4b were defined as AD‐CRC.

### 2.4 CRC panel

We designed the panel with Ion AmpliSeq designer software (Thermo Fisher Scientific) as previously reported.^21^, ^22^ In total, 60 SMGs were selected to establish an in‐house CRC panel (Table S1). These genes were selected according to other reports including TCGA^12^, ^17^ and the Catalogue of Somatic Mutations in Cancer database. In total, CRC panel contained 67,614 amino acids.

### 2.5 Targeted sequencing

Target sequencing was performed with an Ion PGM (Ion Torrent, Thermo Fisher Scientific). Multiplex polymerase chain reaction (PCR) was performed with the CRC panel using the Ion AmpliSeq Library Kit 2.0 (Thermo Fisher Scientific). The library was barcoded, purified, and quantified according to prior report.^23^ Before target sequencing, emulsion PCR was conducted with OT2 200 Kit (Thermo Fisher Scientific).

### 2.6 MMR immune staining

Immunohistochemical analysis (IHC) was performed and deficiency of MMR proteins (*MLH1*, *MSH2*, *MSH6*, and *PMS2*) was judged on formalin‐fixed paraffin‐embedded specimens of endoscopically resected samples for CRA and E‐CRC in accordance with previous report.^23^

### 2.7 Analysis of MLH1 methylation

Methylation‐specific PCR was performed to assess the methylation of *MLH1*with either the MethylEasy Xceed Rapid DNA Bisulphite Modification Kit (Human Genetic Signatures, Sydney, Australia) or the MethylCode Bisulfite Conversion Kit (Thermo Fisher Scientific, MA, USA). The Real‐time PCR and electrophoresis using 2% agarose gel were conducted with the EpiScope MSP Kit (Takara, Shiga, Japan). The primers used in this analysis followed the prior report.^24^, ^25^

### 2.8 Analysis of oncogenic mutation

Each mutation was analyzed whether it was putative‐driver or not according to the OncoKB (http://oncokb.org/).^26^ Oncogenic or likely oncogenic somatic mutations were judged as putative‐driver mutations. Others were judged as putative‐passenger mutations.

### 2.9 Analysis of CRA and E‐CRC data

In CRA and E‐CRC, somatic mutations of the genes involved in the Wnt, MAPK, and cell‐cycle–signaling pathway were sorted according to the MMR status. The following genes were identified as a part of specific pathways: *APC*, *RNF42*, *CTNNB1*, *and AXIN2* for the Wnt signaling pathway; *BRAF*, *KRAS*, *and NRAS* for the *MAPK* signaling pathway; and *TP53*, *ATM*, *and RB1* for the genes of cell‐cycle–signaling pathway.

### 2.10 Analysis of AD‐CRC data

For AD‐CRC, in silico analysis of TCGA data was performed. Somatic mutations of the genes in the Wnt, MAPK, and cell‐cycle–signaling pathways same as CRA and E‐CRC were sorted according to the MSI status. We compared the putative‐driver mutations of Wnt, MAPK, and cell‐cycle pathways among CRA, early CRC, and AD‐CRC in TCGA.

### 2.11 PyClone analysis

For double E‐CRC within same patients (two patients in total), we performed PyClone analysis.^27^ PyClone analysis was conducted to assess cellular prevalence and clonal clusters of each somatic mutation for neoplastic lesion.

### 2.12 Data analysis

The raw data of target sequencing was processed and analyzed with Torrent Suite version 5.0.4. Detailed filter setup was conducted almost the same as previous reports.^22^, ^23^ Sequence data were visually confirmed using the Integrative Genomics Viewer.

### 2.13 Statistical analysis

Fisher's exact test or chi‐square test was used for comparisons between driver mutations in each pathway among cases of CRA, E‐CRC, and AD‐CRC in TCGA. Statistical significance was set at *p* < 0.05, and all statistical analyses were performed using SPSS version 22.0 (SPSS Inc., Chicago, IL, USA).

## 3 RESULTS

### 3.1 Patient characteristics of CRA and E‐CRC

The characteristics of the patients and lesions are summarized in Table 1. In E‐CRC, 25 samples (83.3%) were obtained from ESD. Twenty‐three lesions (76.7%) were IMC. In CRA, 14 samples (63.6%) were obtained from EMR. Ten lesions (45.5%) were high‐grade adenomas (HGA). All CRAs included in this study were tubular adenoma. Oncogenic mutations per each case (median) were two in E‐CRC and one in CRA, respectively. All E‐CRC included in this study was differentiated adenocarcinomas.

**TABLE 1:** Characteristics of patients and lesions.

CRA (15 patients, 22 lesions)	
Age (mean ± *SD*)	69.2 ± 9.4
Gender (Male/Female)	11/4
Samples (EMR/ESD)	14/8
Location (Right/Left)	16/6
Histology^a^ (LGA/HGA)	12/10
Oncogenic mutation per each lesion (median range)	1 (0–4)

E‐CRC (28 patients, 30 lesions)	
Age (mean ± *SD*)	74.7 ± 8.3
Gender (Male/Female)	18/10
Samples (EMR/ESD)	5/25
Location (Right/Left)	9/21
Histology (IMC/SM invasive cancer)	23/7
Oncogenic mutation per each lesion (median range)	2 (1–5)

Abbreviations: CRA, colorectal adenoma; E‐CRC, early colorectal cancer; EMR, endoscopic mucosal resection; ESD, endoscopic submucosal dissection; HGA, high grade adenoma; IMC, intramucosal carcinoma; LGA, low grade adenoma; *SD*, standard deviation; SM, submucosa.

^a^ All CRAs included in this study were tubular adenoma.

### 3.2 Mutations in CRA and E‐CRC

**FIGURE 1:**
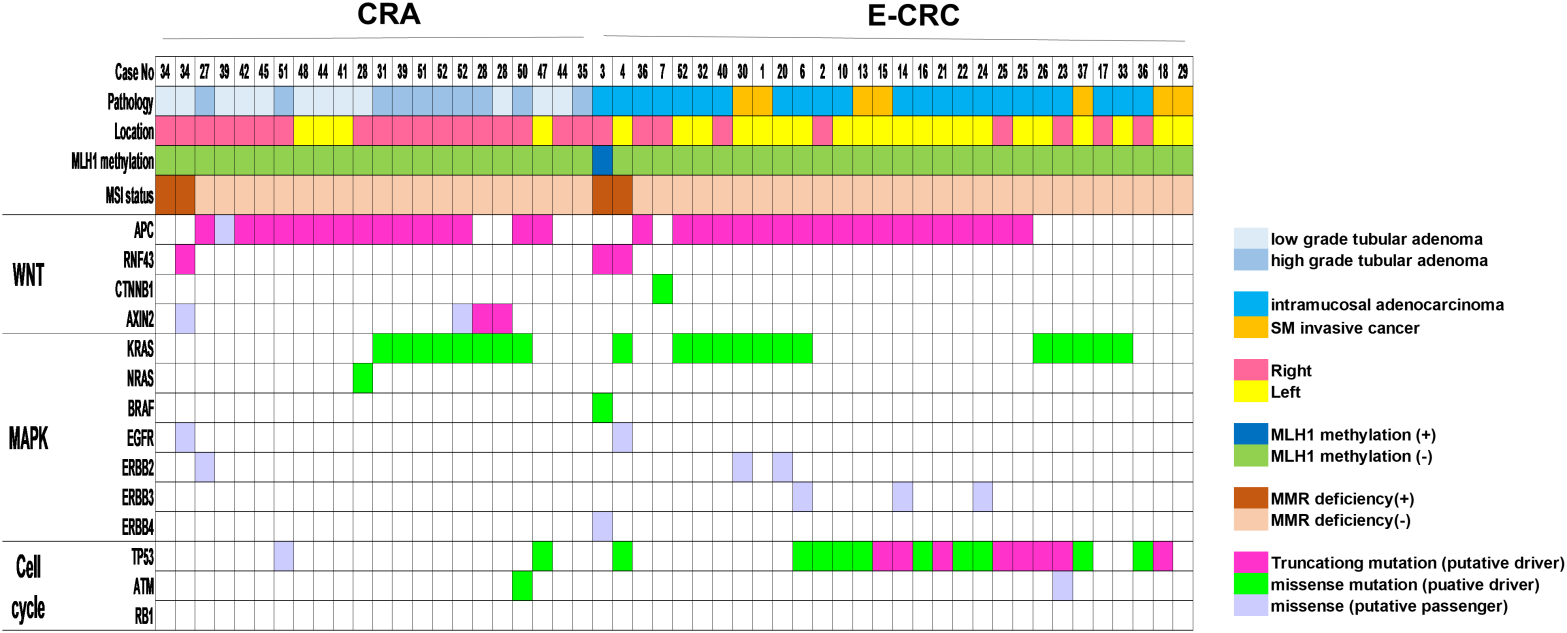
Oncogenic mutation of three pathways (Wnt/ MAPK/ Cell‐cycle) of CRA and E‐CRC. CRA, colorectal adenoma; E‐CRC, early colorectal cancer; MMR, mismatch repair.

Oncogenic mutations of Wnt, MAPK, and cell‐cycle–signaling pathway of CRA and E‐CRC are shown in Figure 1. *RNF43* and *APC* were mutually exclusive. *KRAS*, *NRAS*, and *BRAF* were also mutually exclusive within each case. Almost all of the mutation of *APC* and *RNF43* identified in this study were putative‐driver. *APC* was the most frequently putative‐driver gene in E‐CRC (63.3%, 19/30) and CRA (72.7%, 16/22). In CRA, only one lesion had a putative‐driver mutation of TP53 (4.5%, 1/22) in contrast to E‐CRC. Putative‐driver mutation of *RNF43* was observed in 1 CRA and 2 E‐CRC with MMR deficiency. One case in E‐CRC with an *RNF43* putative‐driver mutation had *MLH1* methylation.

### 3.3 Somatic mutation of AD‐CRC in TCGA

**FIGURE 2:**
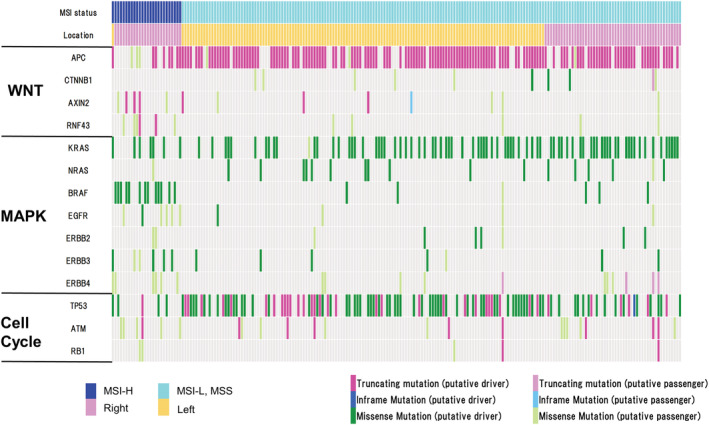
Oncogenic mutation of three pathways (Wnt/ MAPK/ Cell‐cycle) of AD‐CRC TCGA (*n* = 212). AD‐CRC, advanced colorectal cancer; TCGA, Cancer Genome Atlas; MSI, microsatellite instability; MSI‐H, MSI‐high; MSI‐L, MSI‐low; MSS, microsatellite stable.

There were 212 cases of AD‐CRC in TCGA, which are illustrated via an oncoplot in Figure 2. Almost all of the MSI‐high AD‐CRC were located in the right side. In these cases, driver mutations of *CTNNB1*, *AXIN2*, and *RNF43* (i.e., genes of Wnt signaling pathway other than *APC*) were identified. Furthermore, putative‐driver mutations of *BRAF* were frequently observed in these tumors. Conversely, in the MSI‐low and microsatellite stable (MSS) tumors, most of the tumors were located in the left side. Almost all these tumors had a putative‐driver mutation of *APC*. In AD‐CRC, *KRAS*, *NRAS*, and *BRAF* were also mutually exclusive within each case, similar to E‐CRC.

### 3.4 Comparison of the ratio of putative‐driver mutations among the Wnt, MAPK, and cell cycle

The comparisons of the ratios of putative‐driver mutations among the Wnt, MAPK, and cell‐cycle–signaling pathways via Fisher's exact test are shown in Table 2A,B. The ratio of lesions with putative‐driver mutations in all three signaling pathways were not significantly different between AD‐CRC and E‐CRC (22.6% vs. 6.7%; *p* = 0.052). The ratio of lesions with putative‐driver mutations in Wnt and cell‐cycle–signaling pathways were also not significantly different between AD‐CRC and E‐CRC (20.8% vs. 36.6%; *p* = 0.052), but they were significantly different between E‐CRC and CRA (36.6% vs. 4.5% *p* = 0.008). Lastly, the ratio of lesions with putative‐driver mutations in only the Wnt signaling pathway were significantly higher in CRA than in E‐CRC (36.4% vs. 6.7%; *p* = 0.012).

**TABLE 2:** Comparison of putative driver mutations of the Wnt, MAPK, and cell‐cycle–signaling pathways between E‐CRC and AD‐CRC in TCGA.

A. Comparison between E‐CRC and AD‐CRC in TCGA					
Wnt^a^	MAPK^a^	Cell cycle^a^	E‐CRC (*n* = 30) *n* (%)	AD‐CRC (*n* = 212) *n* (%)	*p*‐value
+	+	+	2 (6.7)	48 (22.6)	0.052*
+	+	−	7 (23.3)	44 (20.8)	0.746**
+	−	+	11 (36.6)	44 (20.8)	0.052**
+	−	−	2 (6.7)	23 (10.8)	0.749*
−	+	+	3 (10.0)	20 (9.4)	1.000*
−	+	−	2 (6.7)	16 (7.5)	1.000*
−	−	+	2 (6.7)	8 (3.8)	0.358*
−	−	−	1(3.3)	9 (4.3)	1.000*

B. Comparison between CRA and E‐CRC					
Wnt^a^	MAPK^a^	Cell cycle^a^	CRA (*n* = 22) *n* (%)	E‐CRC (*n* = 30) *n* (%)	*p*‐value
+	+	+	1 (4.5)	2 (6.7)	1.000*
+	+	−	8 (36.4)	7 (23.3)	0.306**
+	−	+	1 (4.5)	11 (36.6)	0.008*
+	−	−	8 (36.4)	2 (6.7)	0.012*
−	+	+	0 (0)	3 (10.0)	0.253*
−	+	−	0 (0)	2 (6.7)	0.502*
−	−	+	0 (0)	2 (6.7)	0.502*
−	−	−	4 (18.2)	1 (3.3)	0.149*

*Note*: +, putative driver (+); −, putative driver mutation (−).

Abbreviations: AD‐CRC, advanced colorectal cancer; CRA, colorectal adenoma; E‐CRC, early colorectal cancer.

^a^ Wnt signaling pathway (*APC*, *RNF42*, *CTNNB1*, *AXIN2*); *MAPK* signaling pathway (*BRAF*, *NRAS*, *KRAS*, *EGFR*, *ERBB2*, *ERBB3*, *ERBB4*); Cell cycle signaling pathway (*TP53*, *ATM*, *RB1*).

* Fisher's exact test.

** Chi‐square test.

### 3.5 PyClone analysis of the double early CRC in the same patients

**FIGURE 3:**
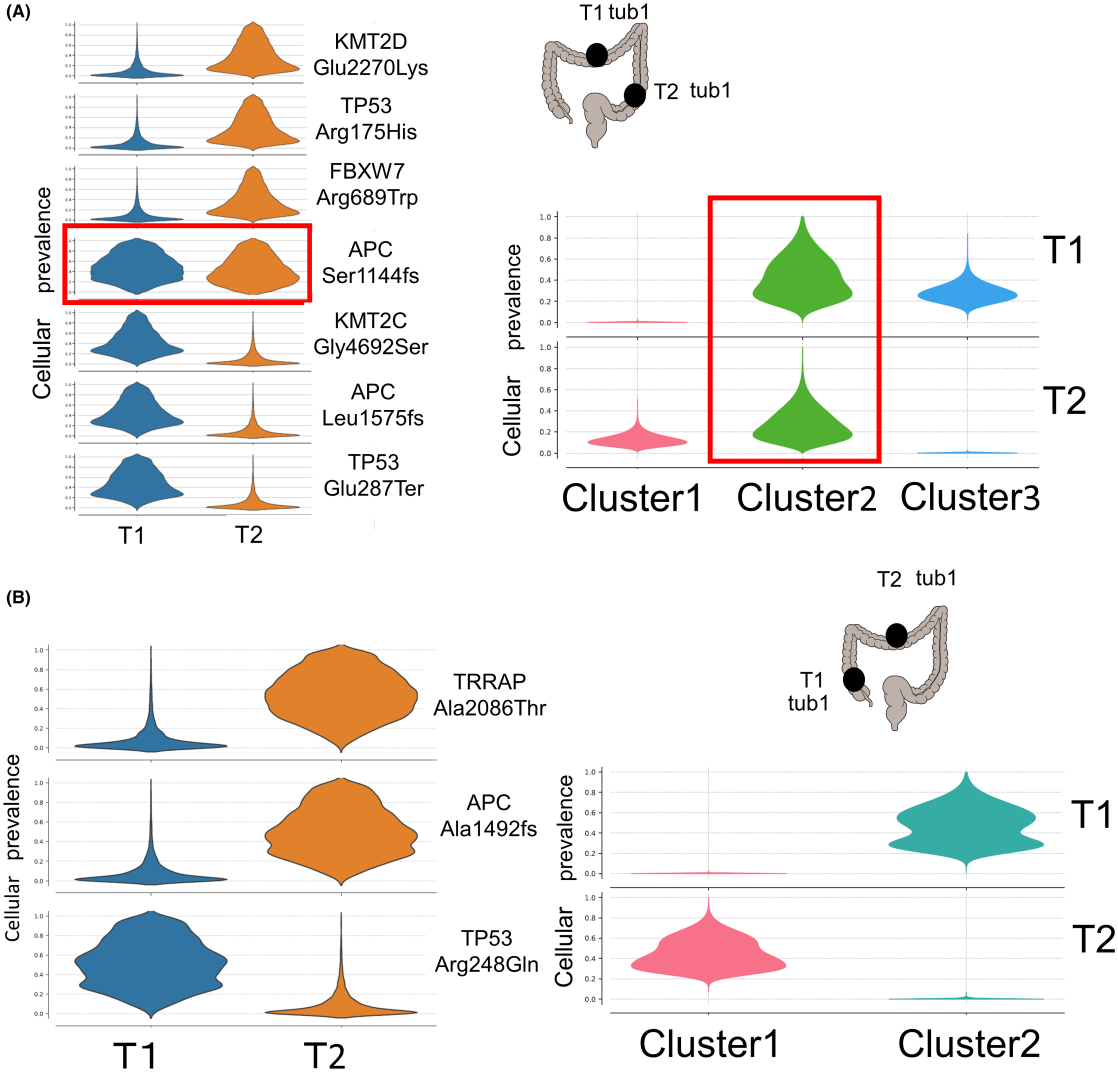
Somatic mutation of double E‐CRC in the same patient analyzed with PyClone analysis. (A) CRC 25. One CRC were located at transverse, while the other was located at descending colon. Both were well differentiated adenocarcinoma. APC Ser1144fs were common in both tumors. Cellular prevalence also showed common cluster (cluster 2). (B) CRC 36. Two CRC were located at transverse colon. Both were well differentiated adenocarcinoma. No mutual somatic mutation or cluster was identified.

There were two patients with double E‐CRC. The results of PyClone analysis are shown in Figure 3A,B. In CRC 25, oncogenic mutations (APC Ser1144fs) were common. Furthermore, cluster analysis based on the PyClone analysis of this mutation was also common. However, in CRC 36, oncogenic mutation and cluster analysis based on PyClone analysis was different.

## 4 DISCUSSION

In this study, we examined the genetic profile of CRA, E‐CRC with clinical samples obtained from EMR, ESD, and AD‐CRC based on TCGA data. We also investigated the validity of Japanese pathological diagnosis for E‐CRC from a genetic aspect. To the best of our knowledge, these points have not been extensively discussed in the literature, and thus our study can be considered novel.

The adenoma–carcinoma sequence is widely accepted as a model of carcinogenesis of CRC.^28^ According to this theory, *APC*, *RAS*, and *TP53* mutations occur for an adenoma to progress to CRC. *APC* is often mutated in tubular or tubulovillous adenomas.^28^ Likewise, in our study, CRA had a putative oncogenic mutation only for the Wnt signaling pathway genes compared to E‐CRC. Furthermore, E‐CRC had a putative oncogenic mutation for the Wnt and cell‐cycle–signaling pathway genes compared to CRA. Additionally, although E‐CRC tended to have more oncogenic mutations only for the Wnt and cell‐cycle‐signaling pathways and less for all Wnt, MAPK, and cell‐cycle‐signaling pathways compared to AD‐CRC, E‐CRC, and AD‐CRC had similar ratios of putative‐driver mutations in each pathway. In another report, Li p et al. reported that APC (77.4%), TP53 (72.9%), and KRAS (53.4%) were mutated in the AD‐CRC dominant cohort.^29^ The mutation rates of APC, KRAS, and TP53 were comparable to those in our E‐CRC and AD‐CRC. Comparing to this report,^29^ E‐CRC in our study also had relatively similar mutational backgrounds to AD‐CRC.

To summarize, it may be true that CRA first obtains driver mutations for the genes of the Wnt signaling pathway (mainly APC) in a typical adenoma–carcinoma sequence. However, in order to progress into an E‐CRC, approximately 40% of tumors may secondarily obtain a driver mutation of *TP53* instead of the genes for the MAPK signaling pathway, unlike in the typical adenoma–carcinoma sequence. And some E‐CRC with oncogenic mutations for the Wnt, cell‐cycling pathway may progress to AD‐CRC by acquiring oncogenic mutations for the MAPK signaling pathway (especially KRAS). These mechanisms seem crucial to comprehensively understand the carcinogenesis of E‐CRC. Based on these results, CRA, and E‐CRC of the Japanese definition are genetically different profiles. E‐CRC, including IMC (which is defined as a high‐grade dysplasia in the WHO classification), already has a genetic background similar to that of AD‐CRC.

In E‐CRC, two cases with MMR deficiency (including one case with *MLH1* methylation) had a putative‐driver mutation of *RNF43*.The two identified mutations of *RNF43* in E‐CRC were both p.Gly659fs (hotspot accounting for 41.7% of alteration^30^, ^31^). Frameshift *RNF43* mutations involving mononucleotide repeats are often observed in colorectal tumors with MMR deficiency.^32^ *RNF43* is an E3 ubiquitin‐protein ligase that reduces the Wnt signaling through the R‐spondin/*LGR5/RNF43* module.^33^, ^34^ Somatic mutations of *RNF43* are identified in over 18% of CRC cases.^31^ Furthermore, across all types of CRC, *RNF43* is a specific mutated gene in the serrated pathway, which is associated with malignancy.^35^, ^36^ In the serrated pathway, *RNF43* often coexists with *BRAF* mutation.^35^ In the present study, one case of *RNF43* somatic mutation co‐occurred with *BRAF V600E* mutation, which is thus a characteristic of the serrated pathway. Interestingly, despite all E‐CRC cases in this study being tubular adenocarcinomas pathologically, we were able to identify their malignant origins from a genetic point of view. In cases of double E‐CRC in the same patient, despite having different locations, some case might have the same driver mutation with same cluster from the viewpoint of cellular frequency, indicating that these two lesions had a common carcinogenic origin.

In AD‐CRC with TCGA, only two cases were presented with putative‐driver mutation of RNF43. It is important to note that in some cases, even in very early‐stage CRC, driver mutations of RNF43 with MMR deficiency can occur and contribute to the neoplastic changes. Still, the rarity of RNF43 mutations in AD‐CRC in the TCGA database is not completely clarified. As for MAPK genes, colonic tumors with BRAF mutations, versus wild‐type tumors, are more likely to be located in the right colon and either be poorly differentiated or be a mucinous adenocarcinoma.^37^, ^38^ In the case of an identified BRAF and RNF43 somatic mutation, despite the tumor being in the right side of the colon, it was an early tubular adenocarcinoma. Thus, the true function of the BRAF mutation in serrated pathway needs to be further elucidated. In metastatic CRC, *KRAS* codon 12/13/61, *NRAS*, and *BRAF* were all mutually exclusive.^39^ Similarly, in E‐CRC, *KRAS* 12/61, *NRAS* codon61, and *BRAF* V600E were mutually exclusive.

This study had some limitations. First, the number of clinical samples was small. Second, our clinical samples might have obtained nontumor cells, as we did not perform microdissection. Third, for we assessed only somatic mutation of DNA, gene expression such as messenger ribonucleic acid or protein was not evaluated.

In conclusion, the definition of E‐CRC according to the Japanese criteria had a different genetic profile from CRA and was more similar to AD‐CRC. Based on the main pathway, it seemed reasonable to classify E‐CRC as adenocarcinoma. The pathological diagnosis of E‐CRC according to Japanese definition seemed to be valid from a genetic point of view.

## AUTHOR CONTRIBUTIONS

**Kenichiro Okimoto:** Conceptualization (lead); writing – original draft (lead); writing – review and editing (lead). **Yosuke Hirotsu:** Data curation (equal). **Makoto Arai:** Data curation (equal). **Kenji Amemiya:** Data curation (equal). **Naoki Akizue:** Data curation (equal). **Yuki Ohta:** Data curation (equal). **Takashi Taida:** Data curation (equal). **Keiko Saito:** Data curation (equal). **Hiroshi Ohyama:** Data curation (equal). **Tomoaki Matsumura:** Data curation (equal). **Motoi Nishimura:** Data curation (equal). **Kazuyuki Matsushita:** Data curation (equal). **Keisuke Matsusaka:** Conceptualization (equal). **Toshio Oyama:** Data curation (equal). **Hitoshi Mochizuki:** Data curation (equal). **Tetsuhiro Chiba:** Writing – review and editing (equal). **jun Kato:** Writing – review and editing (equal). **Junichiro Ikeda:** Writing – review and editing (equal). **Osamu Yokosuka:** Writing – review and editing (equal). **Naoya Kato:** Conceptualization (equal); writing – review and editing (equal). **Masao Omata:** Writing – original draft (equal).

## FUNDING INFORMATION

This report received no sources of external funding.

## CONFLICT OF INTEREST STATEMENT

The authors have no conflicts of interest to declare.

## Supporting information


Table S1.

## Data Availability

Data sharing is not applicable to this article as no new data were created or analyzed in this study.
