# Supplementary material for: Validity of pathological diagnosis for early colorectal cancer in genetic background
Source: Cancer Med. 2023 Feb 3;12(7):8490–8. doi: 10.1002/cam4.5596 (PMC10134368; doi:10.1002/cam4.5596)
Supplement: Supplementary file 1 — Table S1. [file CAM4-12-8490-s001.docx]

| TableS1. Significantly mutated genes contained in colorectal cancer panel |
| --- |
| *MLH1* |
| *MLH3* |
| *MSH2* |
| *MSH3* |
| *MSH6* |
| *PMS1* |
| *PMS2* |
| *APC* |
| *TGFBR2* |
| *MYO1B* |
| *TCF7L2* |
| *CDC27* |
| *FZD3* |
| *MIER3* |
| *TCERG1* |
| *MAP7* |
| *PTPN12* |
| *TP53* |
| *PIK3CA* |
| *SMAD2* |
| *FBXW7* |
| *SMAD4* |
| *SOX9* |
| *ACVR1B* |
| *GPC6* |
| *EDNRB* |
| *ATM* |
| *ARID1A* |
| *NF1* |
| *PTCH1* |
| *KMT2C* |
| *KMT2D* |
| *PTEN* |
| *NTHL1* |
| *RNF43* |
| *SMAD3* |
| *RB1* |
| *AKAP9* |
| *TRRAP* |
| *BRCA2* |
| *SLC9A9* |
| *AXIN2* |
| *TGFBR1* |
| *PIK3R1* |
| *ERBB2* |
| *ERBB3* |
| *ACVR2A* |
| *LRP5* |
| *POLE* codon 97/137/142 |
| *POLD1*codon159 |
| *KRAS* codon 12/13/61/117/146 |
| *NRAS* codon 12/13/61 |
| *BRAF* codon 600 |
| *CTNNB1* codon 32-45 |
| *EGFR* |
| *ERBB4* |
| *CASP8* |
| *CREBBP* |
| *MUTYH* |
| *FAM123B* |
